# Silver Nanoparticles Formation by *Jatropha integerrima* and LC/MS-QTOF-Based Metabolite Profiling

**DOI:** 10.3390/nano11092400

**Published:** 2021-09-15

**Authors:** Afrah E. Mohammed, Lamya Ahmed Al-Keridis, Ishrat Rahman, Modhi O. Alotaibi, Rasha Saad Suliman, Aisha Mohammed Alrajhi, Mudawi M. Elobeid, Monerah R. Alothman, Eman A. Alhomaidi, Shereen M. Korany

**Affiliations:** 1 Department of Biology, College of Science, Princess Nourah bint Abdulrahman University, Riyadh 84428, Saudi Arabia; amoalrajhi@pnu.edu.sa (A.M.A.); shirienmagdy@yahoo.com (S.M.K.); 2 Department of Basic Dental Sciences, College of Dentistry, Princess Nourah bint Abdulrahman University, Riyadh 84428, Saudi Arabia; 3 Department Pharmaceutical Sciences, College of Pharmacy, King Saud bin Abdulaziz University for Health Sciences, Riyadh 11481, Saudi Arabia; sulimanr@ksau-hs.edu.sa; 4 Department of Silviculture, Faculty of Forestry, University of Khartoum, Shambat 13314, Khartoum North, Sudan; emudawi2828@hotmail.com; 5 Department of Biology and Microbiology, Faculty of Science, King Saud University, P.O. Box 22452, Riyadh 11495, Saudi Arabia; Malothman@ksu.edu.sa; 6 Botany and Microbiology Department, Faculty of Science, Helwan University, Helwan 11795, Egypt

**Keywords:** HPLC, antibacterial, cytotoxicity, nano-antibiotics composite, apoptosis

## Abstract

The broad application of metal nanoparticles in different fields encourages scientists to find alternatives to conventional synthesis methods to reduce negative environmental impacts. Herein, we described a safe method for preparing silver nanoparticles (J-AgNPs) using *Jatropha integerrima* leaves extract as a reducing agent and further characterize its physiochemical and pharmacological properties to identify its therapeutic potential as a cytotoxic and antimicrobial agent. The biogenic synthesized J-AgNPs were physiochemically characterized by ultraviolet-visible spectroscopy, dynamic light scattering (DLS), transmission electron microscope (TEM), and energy-dispersive X-ray spectroscopy. HPLC-DAD, followed by LC/MS and the Fourier-transform infrared spectroscopy (FTIR), was applied to detect the biomolecules of *J. integerrima* involved in the fabrication of NPs. Furthermore, J-AgNPs and the ampicillin-nanocomposite conjugate were investigated for their potential antibacterial effects against four clinical isolates. Finally, cytotoxic effects were also investigated against cancer and normal cell lines, and their mechanism was assessed using TEM analysis and confocal laser scanning microscopy (LSM). Ag ions were reduced to spherical J-AgNPs, with a zeta potential of −34.7 mV as well as an average size of 91.2 and 22.8 nm as detected by DLS and TEM, respectively. HPLC GC/MC analysis identified five biomolecules, and FTIR suggested the presence of proteins besides polyphenolic molecules; together, these molecules could be responsible for the reduction and capping processes during NP formation. Additionally, J-AgNPs displayed a strong antibacterial effect, although the ampicillin conjugated form had a very weak antibacterial effect. Furthermore, the NPs caused a reduction in cell viability of all the treated cells by initiating ultrastructural changes and apoptosis, as identified by TEM and LSM analysis. Therefore, J-AgNPs can be formed using the leaf extract from the *J. integerrima* plant. Furthermore, J-AgNPs may serve as a candidate for further biochemical and pharmacological testing to identify its therapeutic value.

## 1. Introduction

Antimicrobial resistance (AMR) is a real threat to humans worldwide. The mortality rate to AMR is continuously increasing and is expected to reach 50 million by 2050 [1]. In addition, bacterial resistance is becoming more prevalent, causing a threat to public health. Thus, alternative therapies are desperately needed to combat the AMR threat. Silver is a popular material for preventing the growth of bacteria, and it has been used for medicinal purposes since ancient times [2]. However, silver causes adverse effects on the human body when used in its natural form; therefore, converting silver to nanoparticles (AgNPs) characterized by a smaller size is necessary. Generally, nanotechnology-based medicine is one of the most promising approaches [3]. Various forms of AgNPs have been widely studied for their antimicrobial potential. AgNPs interact easily with bacterial cell surfaces leading to direct damaging effects via adhesion and penetration, thus disturbing cell components directly or via initiating reactive oxygen species [4] that increase the cell membrane permeability.

Furthermore, AgNPs also take part in neutralizing cell components involved in biofilm formation [5]. AgNPs are also common anticancer agents; they can initiate programmed cell death by disrupting membrane integrity and cellular functions as well as causing damage to the nucleus membrane, genetic mutations, and toxicity [6]. Many scientists are involved in nanotechnology and natural product science to identify novel antibacterial agents [7]. Nanoscience enables the production of various nanoparticles (NPs) from a wide variety of sources, which can be categorized based on their unique chemical, physical, and biological properties [8]. The biofabrication of NPs requires natural sources, including plants, algae, and microorganisms, such as cyanobacteria, eubacteria, and fungi, offering various ecofriendly solutions for pharmaceutical and biomedical applications [9]. The use of biological materials, especially plant extracts and amino acids, enables the easy synthesis of NPs.

In particular, much research has been focused on forming silver nanoparticles (AgNPs) using plants since it has more advantages in relation to other means of synthesis, such as being quick, cost-effective, practical, and environmentally friendly [7,10]. In addition, plants contain diverse phytochemicals and a wide variety of natural organic compounds with complicated chemical structures; such medicinal plants showed great ability in forming AgNPs with desired therapeutic actions [11]. Several studies have also been conducted on AgNPs, and AuNPs synthesized from various medicinal plants, which show the potential of these green synthesized NPs in the delivery of bioactive substances and the overall action of the green NPs for the treatment of Leishmaniasis [10]. An abundance of studies showed the ability of different plant materials to form NPs, and with each type of biosynthesized NPs, spectra of sizes and activities were observed [12,13,14,15]. For example, different studies using plant leaf extracts provided AgNPs with varied sizes and shapes such as cubic, hexagonal, triangular, prismatic, polygonal, spherical, and other shapes. As well as the plant type and origin, besides soil and climate conditions, the quantity and quality of plant components in each extract could be a key factor in contributing to the diverse sizes and shapes of the NPs. It is well understood that the type of phytochemicals present in the extract has a particular influence on the AgNPs size and shape and can suppress the development of certain appearances of the nanocrystal forms of NPs [16]. 

Furthermore, higher plants can produce a significant number of natural products known as secondary metabolites [17], also contributing to the production of new drugs. Consequently, a scope for bioactive compounds in medicinal plants and herbs has gained much attention [18], where considerable research has focused on understanding natural product synthesis, regulation, and function, and various plants have been investigated for their antibiotic and antioxidant abilities, as well as cytotoxic agents, to tackle a variety of pathological conditions [19]. Recent research indicated that more than 80% of the African people depend on medicinal plant materials for effective health care [20]. One of the most efficient genera is Jatropha, which belongs to the Euphorbiaceae [21]. It is widespread in Asia and Africa, especially in tropics and subtropic areas. The genus Jatropha contains around 170 different herbs, subshrubs, shrubs, or even woody trees known for curing various human diseases with a curing ability reaching 80% in Africa, Latin America, and Asia [22]. Jatropha species are a rich source of plant metabolites and active biomolecules with wide-ranging biological activities such as terpenes [23]. It also contains steroids, flavonoids, and diterpenoids [19], which play a significant role as a biologically reducing and stabilizing agent of silver ions that successfully converted to silver nanoparticles [24].

Additionally, certain parts of the Jatropha plant are anti-inflammatory and used to reduce skin inflammation and cure venereal diseases and urinary discharge [25]. Some compounds isolated from this genus, such as coumarins, cyclic peptides, flavonoids, lignans, terpenes, and alkaloids, were identified from the genus Jatropha. Such compounds were characterized by significant properties like antioxidant, anti-insect, molluscicidal, anti-inflammatory, cytotoxicity, antimicrobial, antifungal, and acetylcholinesterase enzyme (AChE) inhibition [26].

Several bioactive compounds were isolated and purified from the widely known medicinal plant *Jatropha podagrica*, including gallic acid, methyl gallate, fraxetin, and tomentin [27]. There is also an increasing concern about *Jatropha integerrima* since it produces two cyclic heptapeptides, integerrimides A and B, which can inhibit neurite outgrowth in neuronal cells and inhibit the growth of human melanoma cells (IPC-298) [28]. Moreover, diterpenoids isolated from *Jatropha integerrima* trunks displayed a more potent inhibitory action of thioredoxin reductase than the curcumin (positive control), indicating significant therapeutic implications for cancer treatment with antioxidant potential and redox balance [29]. Recently, the flower of *Jatropha* *integerrima* has been confirmed as having the highest antioxidant ability amongst 51 edible and wildflowers from China [30], highlighting the abundant natural source of antioxidants [30]. There are reports of other Jatropha species being used for AgNPs synthesis, mainly the *Jatropha gossypifolia* and *Jatropha curcas* [31] but no reports on the biofabrication of AgNPs using *Jatropha integerrima*. In order to study the potential clinical applications of *Jatropha integerrima*, we aimed to investigate its ability as a bio reducing and capping agent for NPs for the first time. Furthermore, possible biomolecules present in plant extract were identified using RP-HPLC-HPLC-DAD, followed by an analytical LC-QTOF-MS beside the FTIR technique. Moreover, biogenic NPs were characterized using UV, DLS, TEM, and EDX, and their biological activities were tested for bacterial growth and cancer cell lines suppression. Furthermore, TEM and LSM were performed for one of the treated cell lines, although this had a low sensitivity for detecting the mechanism behind NPs effectiveness against cancer cell lines.

## 2. Results

The current work aims to examine the significance of *J. integerrima* leaves extract as a biomediator in AgNPs formation and further examination of their antibacterial and anticancer activities. The aqueous extract of *J. integerrima* leaves was used, and the formation of J-AgNPs was recorded first by the appearance of pale yellow to brown color of the mixture of AgNO_3_ and plant extract that increased in intensity in a time-dependent manner; after 24 h, the color was stable. 

### 2.1. Characterization of Biogenic J-AgNPs

**Figure 1:**
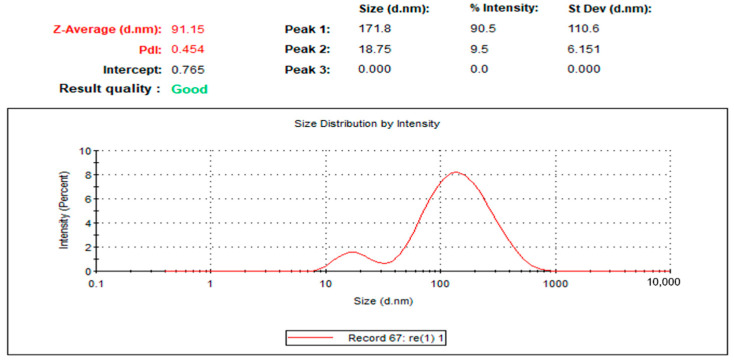
Size distribution of J-AgNPs for three replicates.

**Figure 2:**
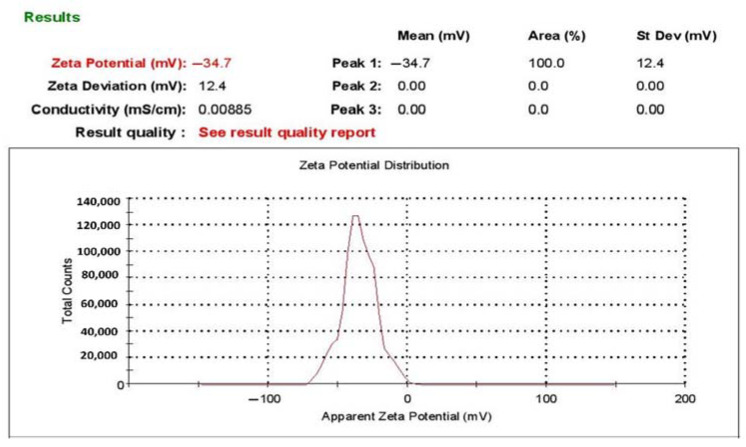
Zeta potential of JAgNPs.

**Figure 3:**
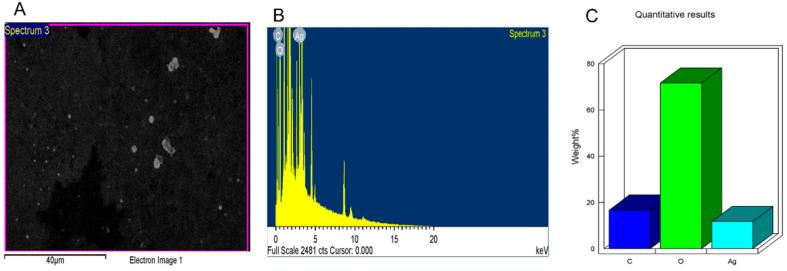
Surface morphology of J-AgNPs (**A**) and quantitative data analysis of images and describing the weights of the carbon, oxygen, and silver atoms using EDS (**B**,**C**).

**Figure 4:**
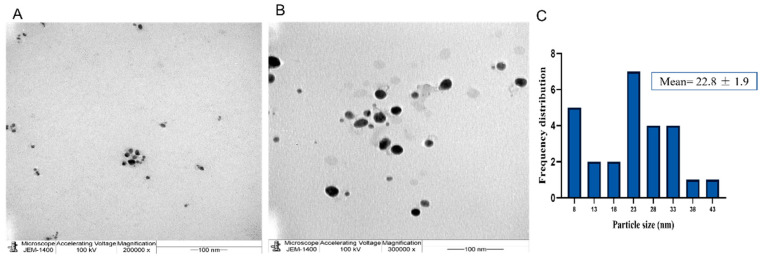
Spherical shape of well-distributed J-AgNPs (**A**,**B**) and their frequency distribution with mean particle size (**C**). Size measurements were analyzed by ImageJ software constructed from TEM micrographs at scale bars: 100 nm.

**Figure 5:**
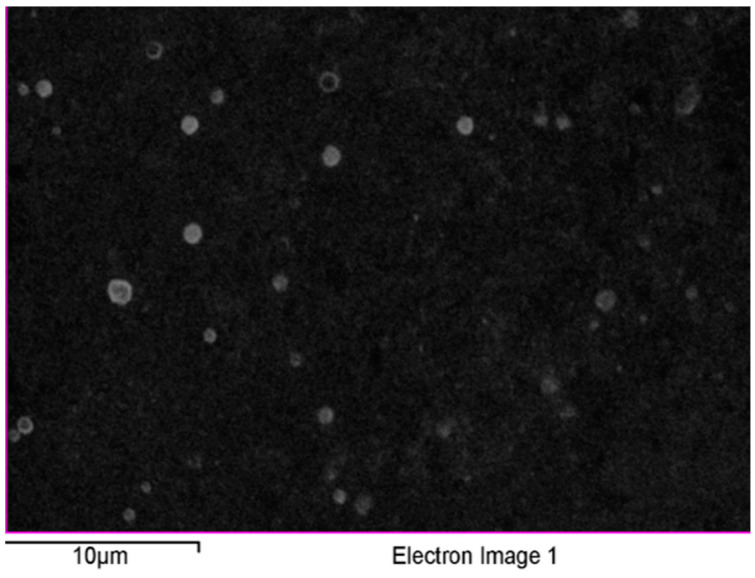
SEM images of J-AgNPs having a clear spherical shape and well-dispersed NPs.

The successful fabrication of silver ions into J-AgNPs using *J. integerrima* leaves extract was confirmed by the UV spectra of NPs at 409 nm, while the zeta sizer and potential measurements showed an average of a 91.51 nm diameter and −34.7 mV, respectively, as shown in Figure 1 and Figure 2. Analysis by EDS verified the existence of the silver element besides the carbon and oxygen originated from the plant extract. The outcomes demonstrated Ag signals at 3 keV (Figure 3) and well-dispersed particles. TEM analysis revealed well-disseminated and uniform nanosized particles with a spherical shape and no significant aggregation (Figure 4A,B), and the NPs size detected was 22.8 ± 1.9 using ImageJ software (Figure 4C). A similar pattern in the NPs morphology was also noted from the SEM image (Figure 5). 

### 2.2. Analytical RP-HPLC and LC–QTOF-MS Identified Compounds

**Figure 6:**
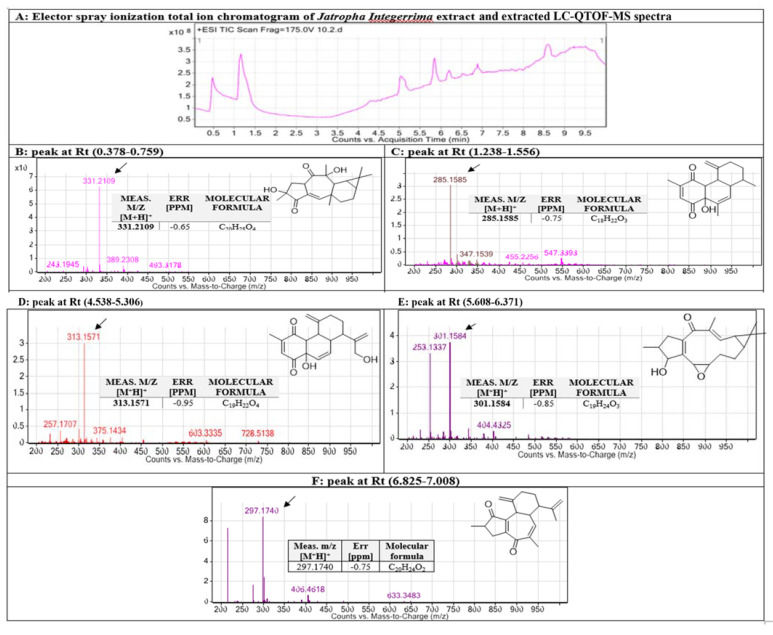
Chemical identification of active metabolites in *J. integerrima* extract using RP-HPLC and LC-QTOF-MS. Base peak chromatogram of *J. integerrima* methanolic extract, extracted LC-QTOF-MS spectra (**A**) and identified secondary metabolites, which are: 2-epi-macroripremyrsinone A (**B**) [32], lathyranes-3 (**C**) [29], and 18-hydroxyjatrophadiketone (**D**) [32], jatrointelone C (**E**), and curcusone A (**F**) [29]. Meas *m/z* implies measured *m/z*. Chemical structures of the tentatively identified compounds re-drawn by chemistry drawing software CehmDraw®.

After conducting a mass assessment (Figure 6), LC-MS records identified the chemical characteristics by the molecular features extraction (MFE) algorithm and the recursive analysis workflow. Information was obtained by screening the identified nodes at different retention times per minute, with the least intensity of 6000 counts that aligned with earlier identified compounds considering adducts ([M+H]^+^ and [M-H]^−^). The identified compounds from the leaves extract of *J. integerrima* are five diterpenoids, namely 2-epi-macroripremyrsinone A, 18-hydroxyjatrophadiketone, lathyranes-3, jatrointelone C, and curcusone A. 

### 2.3. Functional Groups Analyzed by FTIR

**Figure 7:**
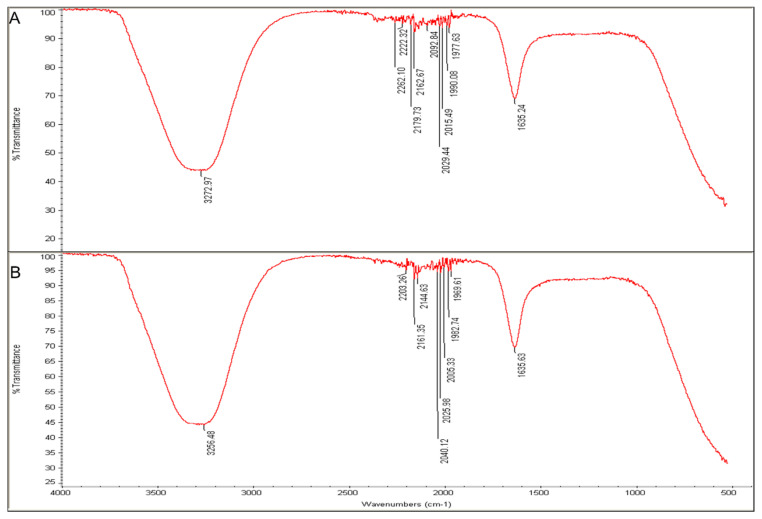
FTIR absorbance peaks of aqueous extracts of *J. integerrima* (**A**) and J-AgNPs (**B**) presenting peaks of the extract organic materials.

Fourier-transform infrared analysis demonstrated the functional groups enveloped the J-AgNPs. The *J. integerrima* leaves extract exhibited spectra peaks at 1635.24, 1977.63, 1990.08, 2015.49, 2029.44, 2092.84, 2162.67, 2179.73, 2222.32, 2262.1, and 3272.97 cm^−1^ (Figure 7A). However, the spectra peaks of J-AgNPs were at 1635.63, 1969.61, 1982.74, 2005.33, 2025.98, 2040.12, 2144.63, 2161.35, 2203.26, and 3256.48 cm^−1^ (Figure 7B). A slight alteration was noted for the absorption bands magnitudes when the spectra of the plant extract were compared with those for J-AgNPs. 

### 2.4. Antibacterial Screening

**Figure 8:**
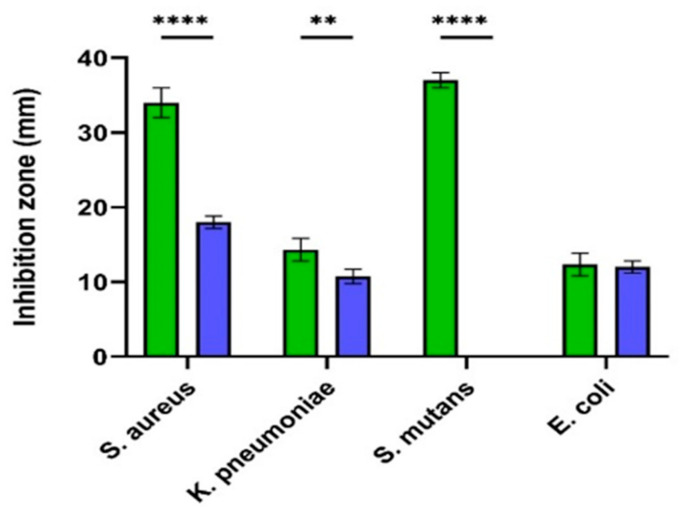
Antibacterial activity of *J. integerrima* AgNPs and Amp-AgNPs. Agar disc zonal inhibition method measuring antibacterial activity as inhibition zones (mm) of J-AgNPs (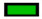) and Amp-AgNPs (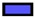) against 4 different bacteria. Two-way ANOVA with multiple comparisons was performed to identify differences between groups. *p* < 0.01 (**), *p* < 0.001 (****).

The antibacterial activity of J-AgNPs (1 mg/mL) was assessed against four (MDR) pathogenic bacteria, including two Gram-positive (*S. aureus* (MRSA) and *S. mutans)* and two Gram-negative (*E. coli* and *Klebsiella pneumoniae*) obtained from BioHouse Medical Lab, Riyadh city, SA, using the agar well-diffusion method. The results were indicated as mm of inhibition zones diameters (Figure 8). Large inhibition zones were noted for J-AgNPs against all tested bacteria; however, no activity was noted for the plant extract alone. The greatest antibacterial activity of J-AgNPs was noted against S. *mutans*, with an average inhibition zone diameter of 37 ± 1.0 mm (*p* < 0.001) followed by *S. aureus*; 34 ± 2.0 mm (*p* < 0.001), *K. pneumoniae*; 14.3 ± 1.5 mm (*p* < 0.01), and lastly *E. coli*; 12.3 ± 1.5 mm (*p* = 0.9926). Furthermore, lower MIC and MBC values were noted for Gram-positive bacteria (0.8 and 1.1 mg/mL, respectively) than Gram-negative bacteria (1.1 and 1.5 mg/mL, respectively), as displayed in Table 1. 

### 2.5. Amp-J-AgNPs Nanocomposites 

**Figure 9:**
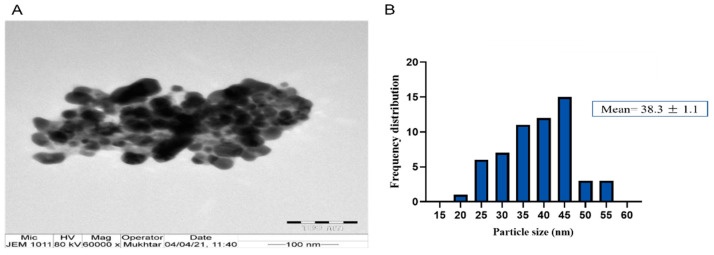
Ampicillin-J-AgNPs nanocomposite (**A**) and their frequency distribution with mean particle size (**B**). Size measurements were analyzed by ImageJ software constructed from TEM micrographs at scale bars: 100 nm.

Currently, a one-pot synthesis approach for ampicillin and J-AgNPs conjugation was performed and detected using TEM. The conjugate image is presented in Figure 8, showing a greater average diameter of Amp-J-AgNPs, indicating that the ampicillin was attached efficiently to the surface of the J-AgNPs, but the structure was drastically altered, identifying large aggregates. On the other hand, Ampicillin antibiotics alone were not active against all the tested microbes. Furthermore, Amp-J-AgNPs nanocomposites displayed lower antibacterial activity than the J-AgNPs alone (Figure 9), indicating the antagonistic effect between the J-AgNPs and Ampicillin.

### 2.6. Cytotoxicity Test

**Figure 10:**
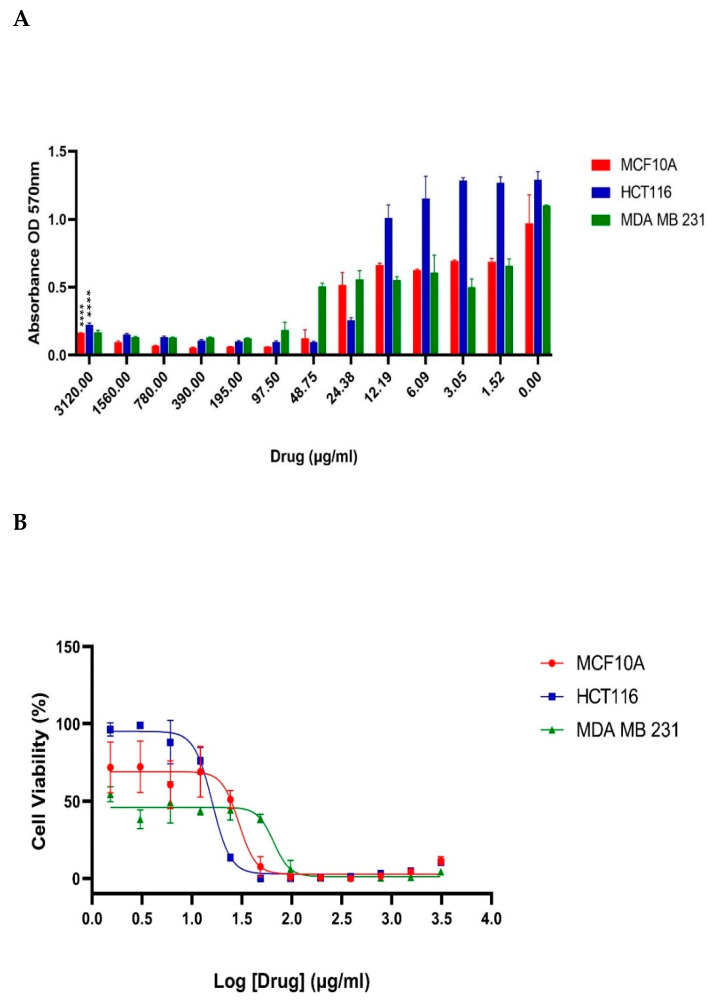
Effect of J-AgNPs on the cell viability of normal and cancer cells. Bar graph showing dose relationship of J-AgNPs on the viability of two human cancer cell lines; HCT116 (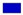) and MDA MB 231 (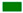) and a normal cell line; MCF 10A (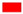). Two-way ANOVA *p* < 0.0001 (****) (**A**). Log dose-response relationship of J-AgNPs on the normalized viability of two human cancer cell lines; HCT116 (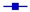) and MDA MB 231 (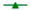) and one normal cell line MCF 10A (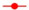). IC50 values of J-AgNPs on each cell line were calculated using log viability vs. normalized response–variable slope (four parameters) (**B**).

The MTT test was performed to identify the cellular metabolic activity of J-AgNPs-treated human cancer cell lines; HCT116 and MDA MB 231, and one normal cell line; MCF 10A (Figure 10). J-AgNPs induced a dose-dependent drop in the cancer cells viability and tested normal cell lines (*p* < 0.0001). A sigmoidal dose-dependent inhibitory response was observed, which best fitted to the model, log (inhibitor) vs. response-variable slope (four parameters) (R^2^ = 0.9203). IC_50_ values were calculated to be 29.53 μg/mL for the normal cell line MCF10A, 16.04 and 65.86 μg/ mL, respectively, for the two cancer cell lines HCT116 and MDA MB 231.

### 2.7. TEM and LSM Analysis of Cancer Cells Treated by J-AgNPs 

**Figure 11:**
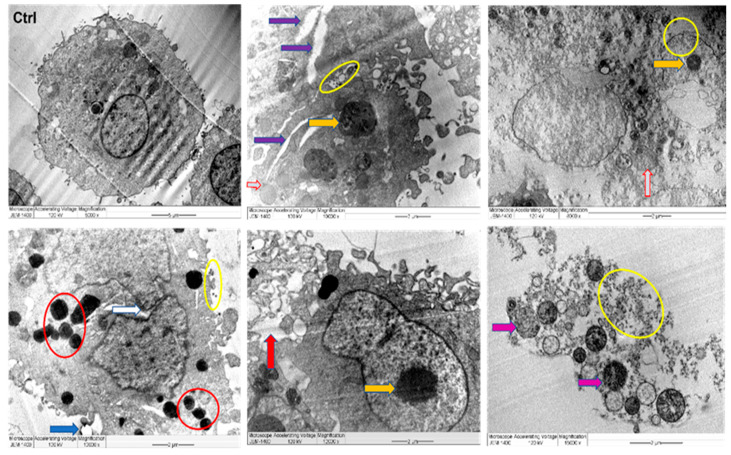
TEM images of MDA MB 231 cell lines, where untreated cell (Ctrl) exhibiting rounded shape cell with complete organelles and nucleus. Image magnification ×6.000; bar 2 μm. Other images at various magnifications show the interaction of NPs and cancer cells leading to ultrastructural changes displaying early apoptosis characteristics such as chromatin condensation and nucleus cleavage (white arrow) and over whole-cell shrinkage as well as late apoptosis: lipid droplet (blue arrow), peroxisomes (red circle) and enlarged mitochondria (grey arrow), damaged mitochondria (violet arrow), and condensed nucleus (yellow arrow). In addition, damaged cancerous cells are observed with NPs located in the cytoplasm, outer cell, and nucleus membranes (yellow circle).

Untreated MDA MB 231 cells displayed distinct tumor cell features such as integral cell membrane, clear microvilli, and large nucleus bound with compact nuclear membranes. Furthermore, cellular organelles were well dispersed in the cytoplasm, and some vacuoles were disseminated. However, the morphology of the J-AgNPs-treated MDA MB 231 cells were distinctly altered when compared against the control (untreated MDA MB 231 cells). The TEM depicts that J-AgNPs are scattered throughout the cytoplasmic membrane and organelles of the treated cells with no NPs observed inside the enlarged nucleus. In addition, the cells appeared to have a deformed membrane appearing leaky, and cellular debris was found scattered around the cell with the disappearance of microvilli. Furthermore, damaged nucleus, large vacuoles, lipid droplets, and the numbers of peroxisomes were evident, and the irregular nucleus membranes were observed (Figure 11).

**Figure 12:**
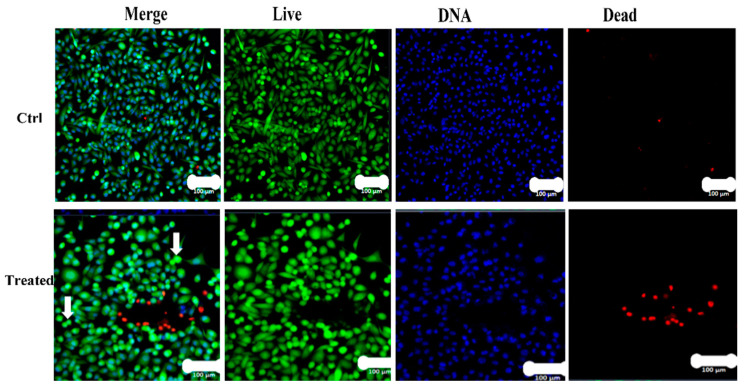
Confocal LSM imaging of J-AgNPs treated MDA MB 231 cell lines. MDA MB 231 cells were treated with either buffer (Control) or J-AgNPs. Cells stained with Calcein AM (Green), propidium iodide (Red) and HOECHST33342 (Blue). Overlay of all three stains (Merge). A representative of 6 different fields of view is shown. Cells that had a bright green fluorescence were live cells. Cells with a bright blue color indicated fragmented nuclei involving concentrated chromatin and were identified as apoptotic cells. Colonies of apoptotic cells were seen in the treated images, and many round stressed cells were scattered throughout the image (white arrows).

Additionally, MDA MB 231 cells were stained with Hoechst 33342 to evaluate apoptotic features. Apoptotic bodies were detected, as shown by the bright blue fragmented nuclei. The control and healthy cells had significant integral nuclei (Figure 12). After 24 h of J-AgNPs exposure, MDA MB 231 showed an increment in apoptotic bodies and reduced cell viability.

## 3. Discussion

Nanotechnology in upgrading human health via medical applications has recently gained immense interest. The application of biological material in nanotechnology is a new and unique ecofriendly technique; yet, insufficient investigations have been carried out so far. The current study used a biogenic agent (*J. integerrima* leaves extract) as a biomediator for the first time for AgNPs formation, and successful conversion was achieved and approved using different approaches.

During the characterization of NPs, the UV absorption at 409 nm indicated that the NPs had a spherical structure; this was later confirmed by TEM analysis. In addition, UV and TEM analysis revealed a similar observed trend to those reported by Mohammed et al. [12] for AgNPs prepared by different plant extracts. The spherical shape of the J-AgNPs might have a higher surface-to-volume ratio compared with NPs from other plant leaf extracts that formed cubic, hexagonal, and other nonspherical shapes as indicated by Xu et al. [33]. 

The size distribution detected by TEM indicated well-dispersed NPs and no particle-to-particle adherence, which might be due to the capping ability of the plant biomolecules resulting in repulsion among the NPs. Although TEM and DLS indicated a nanoranged size for J-AgNPs (22.8 and 91.15, respectively), varied diameters were noted, which might be accounted for the difference in both techniques. DSL analysis for hydrodynamics showed larger J-AgNPs size than those noticed by TEM analysis, which might be related to the existence of plant biomolecules on the J-AgNPs surface. It could also be related to the NPs aggregation and dispersion in the solution used in DSL, and the NPs expected interaction and TEM analysis gave the estimated surface area of the transmitted electrons [34]. Negative zeta potentials were noted in many reports that studied biogenic NPs, which might have resulted from negatively charged biomolecules that cover NPs; therefore, repulsion among particles is expected and provides stable NPs [35]. The EDS for J-AgNPs surface analysis showed 3 keV for Ag and 1 keV peaks for C and O, indicating that plant biomolecules were utilized for NPs formation. The peak at 3.1 keV noted by the EDS spectrum for Ag was previously confirmed by Alqahtani et al. [2], who had prepared AgNPs using lichens.

Additionally, El-Naggar et al. [36] noted that the biomolecules found in the cell extract could be behind the reduction process of Ag ions. Therefore, in an attempt to detect which biomolecules were present in *J. integerrima* leaves that functioned as reducing and capping agents for NPs, HPLC at different *m/z* values and retention times and FTIR were utilized. For the plant-extract biomolecules identified by HPLC, the detected *m/z* value at retention time (0.378–0.759) well correlated with the parent compound 2-epi-macroripremyrsinone A [31] with *m/z* [M+H]^+^ 331.2109 daltons and [C_20_H_26_O_4_]^+^ molecular formula, in positive ion mode, and [M-H]^−^ with *m/z* 329 daltons in negative mode, demonstrating a molecular weight of 330 gmol^−1^ for that compound. The determined *m/z* value at a retention time (1.318–1.556) correlated with the parent compound lathyranes-3 [29]. 15-a cetoxy-5,6-epoxylathyr-12-en-3-vol-14-one with *m/z* [M+H]^+^ 319.2429 daltons and a molecular formula of [C_20_H_28_O_3_]^+^, in positive ion mode, and [M-H]^−^ with *m/z* 317 daltons in negative mode highlighted a molecular weight of 318 g mol^−1^ for that compound. Detected *m/z* value at retention time (4.538–5.306) well correlated with the parent compound 18-hydroxyjatrophadiketone [32] with *m/z* [M+H]^+^ 315.1571 daltons and a molecular formula of [C_19_H_22_O_4_]^+^, in positive ion mode, and [M-H]^−^ with *m/z* 313.234 daltons in negative mode, representing a molecular weight of 314 g mol^−1^ to the compound. The value of *m/z* at retention time (5.608–6.371) well correlated with the parent compound jatrointelone C [29] with *m/z* [M+H]^+^ 301.1584 daltons and a molecular formula of [C_19_H_24_O_3_]^+^, in positive ion mode, and [M-H]^−^ with *m/z* 299 daltons in negative mode, demonstrating a molecular weight of 300 g mol^−1^ to that compound. The appeared *m/z* value at retention time (6.825–7.008) were correlated with the parent compound curcusone A [29] (with *m/z* [M+H]^+^ 297.1704 daltons and a molecular formula of [C_20_H_24_O_2_]^+^, in positive ion mode, and [M−H]^−^ with *m/z* 295 daltons in negative mode, showing a molecular weight of 296 g mol^−1^ to that compound. 

In the current study, five diterpenoids were separated and identified from the aqueous extract of the *J. integerrima* leaves, which were 2-epi-macroripremyrsinone A, and 18-hydroxyjatrophadiketone, lathyranes-3, jatrointelone C, and curcusone A. Diterpenoids are synthesized via the HMG-CoA reductase pathway in plants, animals, and fungi. They consist of a basic structure of four isoprene units, and most have a long hydrocarbon chain (C_20_H_32_) structure. In nature, they are used in the formation of many biologically important compounds such as chlorophyll, quinones, and vitamin A and E. Diterpenoids are recognized for having several valuable medicinal attributes, such as being antimicrobial and anti-inflammatory, and some are used as chemotherapeutic agents, such as the taxanes, useful in the treatment of a variety of cancers including breast cancer [37]. A review published in 2013 had characterized diterpenoids from Jatropha, listing and structurally characterizing all the identified diterpenoids [22]. Diterpenoids can be classified based on their basic skeleton structure; seven have been characterized: tigliane, casbene, daphnane, lathyrane, Jatropha, and podocarpane and rhamnofolane [22]. 18-hydroxyjatrophadiketonehydroxyl has a podocarpane skeleton and is similar to 18-hydroxyjatrophadiketone, curcusone A has a basic skeleton structure of daphnane, and Jatrointelone C has a basic skeleton structure of a rhamnofolane. An earlier report about the chemical constituents of essential oils extracted from both seeds and leaves of *J. integerrima* showed many different aliphatic, aromatic, and monoterpenes hydrocarbons. The only diterpene identified was (E)-Phytol, found in the plant’s leaf [38]. A more recent study identified five new diterpenoids from *Jatropha curcas*, two of which were those identified in the present study: 2-epi-macroripremyrsinone A and 18-hydroxyjatrophadiketone. Both diterpenoids did not display any cytotoxic properties in a human prostate cancer cell line (PC-3) as assessed by CCK-8 assay even at high concentrations of 20 μM [32]. Jatrointelone C and curcusone A displayed potent cytotoxic activity (IC_50_ = 16.3 ± 1.3 and 6.8 ± 0.5 μM, respectively) as assessed by inhibition of thioredoxin reductase activity using the DTNB reduction assay [29]. Lathyranes-3 was previously identified from *Euphorbia ebracteolata* Hayata and indicated no cytotoxic activity against human promyelocytic leukemia, hepatocellular carcinoma, lung cancer, breast cancer, and colorectal cancer cell lines [39]. 

Furthermore, the surface functional groups were detected by FTIR and the spectra peaks noted for plant extract allocated to different functional groups were also noticed for J-AgNPs solution. The major detected peaks from both tested materials were 1635.24 and 3272.97 cm^−1^ for plant extracts and 1635.63 and 3256.48 cm^−1^ for J-AgNPs specified for amides involving proteins. The bands at 3272.97 and 3256.48 cm^−1^ are allocated for NH (amide) in proteins peptide bond and OH stretching for polyphenolic. The absorption peaks at 1635.24 and 1635.63 cm^−1^ were assigned to proteins (amine groups) and alkenes (C=C stretching vibrations) [40,41]. Bands were observed at 1977.63 and 1990.08 for C-H bending (aromatic compounds), 2015.49, 2029.44, 2092.84, N=C=S stretching (isothiocyanate), 2162.67, 2179.73, S-CΞN stretching (thiocyanate), and 2222.32, 2262.1 CΞC stretching (alkyne). Based on the presented peaks, the presence of protein and polysaccharide in both tested materials (major peaks) suggested their contribution in the process of Ag ions reduction leading to the formation of J-AgNPs capped with biomolecules that prevent their aggregation. Similar findings were also noted [13,42]. The shift in peaks transmittance of FTIR signals for J-AgNPs concerning plant extracts might be due to probable interactions between functional groups (capping and reducing agents) and NPs or metal ions [43]. Such observed shifting suggests the contribution of aromatic compounds, polyphenolic, and proteins in the bioreduction process [44]. Results from HPLC and FTIR indicated that 2-epi-macroripremyrsinone A, 18-hydroxyjatrophadiketone and lathyranes-3, jatrointelone C, and curcusone A, besides polyphenolic aromatic compounds and proteins, could be involved in the reduction process of Ag ions to J-AgNPs and could have also acted as capping agents resulting in stable NPs with no clear agglomeration.

Additionally, the antimicrobial activity of J-AgNPs was confirmed in the current study. The widespread use of antibiotics has resulted in many resistant bacterial strains; therefore, there is a need to develop newer drugs with a preference toward using natural plant-based products. Using plant extracts for AgNPs formation could also be a feasible approach in microbial mitigation since their synergistic effect is expected. Jatropha is a genus of plants known for its medicinal properties [45]. Different Jatropha species have been earlier stated for their antimicrobial effect against many bacteria [46,47]. To our information, the present research stands as the first report that uses *J. integerrima* species for AgNPs formation. It was noted that plant extract showed no activity against tested microbes, suggesting that 2 mg/mL was a low concentration for antimicrobial effect. However, J-AgNPs was found to display the strongest antimicrobial potential against the Gram-positive bacteria (*S. mutans* and *S. aureus*) and approximately 50% less activity on the Gram-negative bacteria (*K. pneumoniae* and *E. coli*). MIC and MBC results also indicated that lower J-AgNPs concentrations were needed to suppress the growth of Gram-positive bacteria compared to those needed for Gram-negative bacteria. It was expected that the J-AgNPs would have easy dispersion in Gram-negative more than in Gram-positive bacteria due to the bacterial cell wall structure. However, our findings contradict this hypothesis, although the current observations could come about if specific resistance strains were tested in this study. However, our findings suggested a potential effect for J-AgNPs against both bacterial types. The efficiency of biogenic J-AgNPs as an antibacterial agent could be associated with their ability to enhance cellular oxidative stress leading to cell structure and biomolecules destruction and cell death [4,48]. 

On the other hand, in experimental medicine, it is useful to test AgNPs-antibiotic conjugates to mitigate microbial resistance to antibiotics; therefore, the ampicillin–J-AgNPs nanocomposite was tested. We found the conjugate to confer less antimicrobial activity than the nonconjugated form, which was highly significant in the Gram-positive-tested bacteria. Thus, it is clear that conjugation with ampicillin caused a spectrum of antimicrobial antagonism of the J-AgNPs, ranging from 0% to 100% antagonism, where the most significant level of antagonism is found in the Gram-positive bacteria and least in the Gram-negative bacteria. Further studies on a larger panel of Gram-positive and Gram-negative bacteria will help identify if the pattern of activity and antagonism is significant when compared with the Gram-positive and negative types. Our findings disagree with many reports that showed enhanced antimicrobial activity of ampicillin or other antimicrobial conjugated forms of either gold or silver nanoparticles [49,50,51,52]. It could be speculated that since the ampicillin and J-AgNPs had clustered together, forming large aggregates, this might cause the lack of any synergistic antimicrobial effect and impaired amp-J-AgNPs antimicrobial activity. It is well known that a large surface area is one of the characteristic features of nanoparticles known to augment antibacterial activity because the small size enhances the easy penetration inside microbial cells [53]. 

Many reports in the literature described contradictory observations of the antimicrobial activity of different antibiotic conjugated forms of NPs. For example, AgNPs inhibited the growth of *S.* *typhimurium* when combined with particular antibiotics, tetracycline, neomycin, kanamycin, and enoxacin; however, such an activity was not noted when AgNPs were conjugated with penicillin and ampicillin [54]. It seems some antibiotics enhance the binding ability of AgNPs onto the bacterial cell surface; hence, a synergistic effect is observed, but other antibiotics such as ampicillin may reduce such an ability. It is expected that NPs have antimicrobial activity against resistant microbes; however, the reduction in activity when conjugated with antibiotics could also be related to a weak interaction and capping capacity of antibiotics surround the AgNPs core, which might be the case for ampicillin.

Furthermore, classical cancer medications have significant side effects; thus, looking for alternatives is needed. However, AgNPs is a known agent in cancer treatment. In the current study, further biological investigation of J-AgNPs was evaluated against cancer cell lines to provide a fundamental approach in cancer therapy. The cytotoxic effect was assessed, indicating efficient NPs in cancer cell mitigation. A substantial body of evidence from earlier investigations identified several plants and their products to have anti-proliferative and cytotoxic properties, and thus, such natural plants and their derivatives could potentially be beneficial as cancer medications. In the current study, the MTT cytotoxicity assay was performed to determine the cytotoxic effect of J-AgNPs on two human cancer cell lines and a normal cell line. MDA MBA 231 is a triple-negative (ER-, PR-, and HER2-) human epithelial breast cancer line, and HCT116 is a colorectal cancer cell line. MCF 10A is a nonmalignant human breast epithelial cell line used as a control. J-AgNPs displayed a cytotoxic effect on both cancer lines as well as the normal cell. The IC_50_ value was lowest for the colon cancer cell line (16.04 μg/mL) but was greatest for the breast cancer cell line (65.86 μg/mL), whereas the IC_50_ at the normal cell was 29.53 μg/mL. Therefore, the colorectal cancer cell line could be differentially targeted since a lower concentration of NPs was required to kill 50% of the cells. Our results indicated that the J-AgNPs are not differentially cytotoxic to all cancer cells, especially in the case of breast cancer; there would be no differential cytotoxic effect to the metastatic breast cancer cells; instead, a significant cytotoxic effect would occur in normal breast epithelial cells. It is important to note that very few numbers of cancer cells and normal cells were tested; therefore, the conclusions from the current results may not reflect the true cytotoxic therapeutic potential of J-AgNPs. Thus, it is necessary to screen a large panel of normal and cancerous cells to identify the therapeutic potential of J-AgNPs precisely.

Other studies confirmed that J-AgNPs indeed have cytotoxic properties. One particular study used two different *Jatropha* species to separate AgNPs using the aqueous extract from the stem of the plants. MTT assays were performed on a lung cancer cell line (A549), and IC_50_ values were 19.5 μg/mL for *Jatropha curcus* and 13.5 μg/mL for *Jatropha gossypifolia* [31]. The plant’s root may have more cytotoxic properties, as noted for the *J. curcas* [45]. In another study, *J. curcas* root showed potent cytotoxic activity against human colon adenocarcinoma (HT-29) cells, but also a similar degree of cytotoxicity was observed in hepatocytes [55]. *Jatropha gossypiifolia* latex part showed strong genotoxic effects [56]. The latex part of the *J. integerrima* plant was also shown to contain cycloheptapeptide with strong cytotoxic properties in human nasopharyngeal carcinoma cells [57]. The literature suggests that the Jatropha plant is cytotoxic and has anti-inflammatory properties [55] but may also be genotoxic, especially the latex part. In the current study, the plant leaves were used, and although five components of the leaves extract were separated and identified, they were not individually assessed; thus, it can be reasonably presumed that the active components of *J. integerrima* together in the form of silver nanoparticles displayed cytotoxic effects in cancer cells and the normal cell. Further studies utilizing specific parts of the plant and preparing AgNPs with purified identified components from each part would help to better understand the properties of *J. integerrima* and its utilization as AgNPs for anticancer cytotoxic therapy.

In a trial to detect the possible mechanism behind their cytotoxic effect, J-AgNPs treated cells were detected using TEM. Noticeable ultrastructural changes were visualized in MDA MB 231 cells using TEM. The damaged cells were observed with J-AgNPs located in the cell membrane and cytoplasm. In addition, cell apoptosis features were seen; fragmented nucleus, irregular nucleus membrane, lipid droplet, large vacuoles, low numbers of microvilli, and irregular general cellular shape. The observed changes could be related to the small J-AgNPs size, enhancing their interaction with cells due to a higher affinity and easy penetration into the cellular structures, thus inducing cell death. However, it might also be related to the biomolecules coating the J-AgNPs that enhanced their incorporation [58]. Cell structural changes and damage could also be related to the oxidative stress induced by J-AgNPs [59,60]. Due to their role in reactive oxygen species (ROS) metabolism [61], peroxisomes numbers were increased in the treated cells, which could be a cellular response to reduce the oxidative stress caused by the J-AgNPs treatment.

Furthermore, metastatic properties of cancer cells might be altered due to their interaction with J-AgNPs resulting in plasma membrane damage and microvilli reduction [62]. Similar apoptotic features were noted for magnetic iron oxide NPs loaded with the antitumor drug doxorubicin treated human monocytic THP-1 cells [63]. Successful insertion of J-AgNPs inside cancer cells and located in various cell organelles. Further investigations on the treated cell lines were undertaken using LSM images, which showed a reduction in cell viability and enhancement of apoptotic bodies. Cell damage was noted by the Hoechst 33342 stain that easily attaches to DNA fragments from the apoptotic cell providing blue fluorescence [64]; therefore, the apoptotic features were clear from the DNA fragmentation and nuclear condensation due to the blue color noted in the LSM images. Apoptotic cells contain split nuclei with dense chromatin; however, the late apoptotic and necrotic features have been known to have DNA stained with red color PI [65]. Colonies of apoptotic cells can be seen throughout the J-AgNPs-treated cell, identified as clear spherical structures, indicating cellular stress. Our findings are similar to those of previous reports when colon cancer cells (HCT-116) were treated with a hyaluronic acid layered lipid-based chimeric nanoparticle and recorded by confocal LSM [65]. From the information presented, it is reasonable that the mechanism of cytotoxicity is the interference and interaction of NPs directly with the cell wall and organelles leading to disturbance and leakage and, consequently, dysfunction and cell death. However, it could also be suggested that J-AgNPs enhance ROS production, damaging cell biomolecules, leading to cell structural changes, dysfunction, and apoptosis. It could also be postulated that J-AgNPs released Ag^+^, leading to oxidative stress and DNA destruction, resulting in cellular damage that could be demonstrated as apoptosis [66].

## 4. Materials and Methods

### 4.1. Plant Material

*Jatropha integerrima* leaves were obtained from the nursery of the royal commission for Riyadh city, Riyadh, Saudi Arabia, during November 2020 from a 7 month plant. *J. integerrima* is a cultivated plant native to Cuba; however, it is well adapted and widely spread in Riyadh city gardens, parks, and urban areas. Collected plant identities were verified and determined at Princess Nourah Bint Abdulrahman University Herbarium, Riyadh, Saudi Arabia, by the plant taxonomist Dr Najla Alshaye, Department of Biology, College of Science, Princess Nourah Bint Abdulrahman University, and Dr Yahya Masrahi, Department of Biology, Faculty of Science, Jazan University, Jazan, Saudi Arabia, and then stored in polythene bags at 4 °C before usage. Leaves were rinsed with distilled water, air-dried, and milled to a fine powder employing the IKA-Werke milling machine (GMBH and Co., Staufen im Breisgau, Germany). Plant materials were stored in sealed plastic bags at room temperature for further usage. 

### 4.2. Aqueous Extracts for AgNPs Synthesis 

Aqueous leaves extract was prepared by adding the powder to distilled water at a ratio of 2:100 (*w:v*). First, the combinations were subjected to heat treatment (10 min, 80 °C) and then filtered via Whatman filter paper at a diameter of 125 mm. Subsequently, 10 mL of filtrate was added to 90 mL of 1 mM AgNO_3_ in a flask and heated for 15 min at 80 °C. Then, the reaction was kept in the dark at room temperature for 24 h until the dark color was stable. Subsequently, the combination was centrifuged for 15 min at 14,000 rpm, the supernatant was removed, and the pellet was subjected to distilled water two times for washing at the same conditions and then aspirated into a glass plate to enable drying at room temperature. Finally, a sample of J-AgNPs was ready for analysis at a 1 mg/mL concentration for further investigations.

### 4.3. Characterization of J-AgNPs

Various approaches were undertaken to characterize the biogenic J-AgNPs prepared in the present investigation, such as:

#### 4.3.1. Ultraviolet-Visible Spectroscopy

After the color of the reaction medium (*J. integerrima* extract and AgNO_3_) was stable at dark brown, ultraviolet-visible (UV-Vis) spectroscopy absorption was assessed by a spectrophotometer (BIOCHROM Libra S60PC, Serial Number: 119377, England). Assessments were taken after a 24 h incubation of the mixture within the range of 300–600 nm, and the plant extract was used as a blank [41].

#### 4.3.2. Hydrodynamic Size and Surface Area Analysis

The pattern of the size distribution and zeta potential were assessed by a dynamic light scattering (DLS) system by a Zetasizer (NANO ZSP, Malvern Instruments Ltd., Serial Number: MAL1118778, version 7.11, Malvern, UK) [67].

#### 4.3.3. Size Distribution and Morphology Analysis Using Transmission Electron Microscopy (TEM)

The J-AgNPs size distribution and morphology were investigated by TEM (JEM-1011, JEOL, Tokyo, Japan) at 80 kV voltage. A drop was taken from J-AgNPs solution and located on a carbon-coated copper grid (200 mesh) and dried in a vacuum desiccator [68].

#### 4.3.4. Energy-Dispersive X-ray Spectroscopy (EDS)

Scanning electron microscope (SEM) (JEOL, JED-2200 series, Tokyo, Japan) supplied with energy-dispersive X-ray spectroscopy (EDS) was used for surface analysis of NPs and confirmation of the presence of silver element accurately [69].

### 4.4. Analysis of the Plant Extracts by Analytical RP-HPLC Method

Methanol (HPLC grade) and formic acid were obtained from Honeywell (Seelze, Germany) and Sigma-Aldrich (St. Louis, MO, USA), respectively. The plant extract was inserted in the Agilent1260 Infinity HPLC system (Markham, ON, Canada) accompanied by diode-array detection (DAD) detector. The separation process was undertaken in a reverse-phase mode applying phenomenex kinetex-C18 column (4.6 mm × 250 mm, 5 μm) with elution gradient; 0–1 min, 5% B; 1–11 min, 5–100% B; 11-13 min, 95% B; 13–15 min, 5% B; 15–16 min, 5% B using 0.1% HCOOH in water and 0.1% HCOOH in ethanol as mobile phase A and B, respectively. The material was inserted at a flow rate of 1 mL/min. The DAD-collected UV spectrum at 200, 225, 250, 275, 300, 325, and 350. ChemStation software open LAB CDS A.02.02 Edition (Wilmington, DE, USA) was used for data processing.

### 4.5. C–QTOF-MS Method

Extract analysis was performed by the Agilent1260 Infinity HPLC system combined with Agilent 6530 quadrupole time of flight (Markham, ON, Canada). Agilent extend-C18 column (2.1 mm × 50 mm, 1.8 μm) was used for separation, applying the same elution gradient for RP- HPLC analysis. A volume of 10 µL was inserted at a flow rate of 300 µL/min. The MS1 acquisition technique was attained at 100–600 *m/z* mass in positive mode. The mass spectrometer was set at 300 °C, and 8 L/min as gas temperature and gas flow rate, respectively; 35 psig for Nebulizer; 350 °C and 1 L were SheathGas temperature and SheathGas flow rate, respectively. Agilent MassHunter, qualitative analysis software (Wilmington, DE, USA) was used for MS1 data. 

### 4.6. Analysis of Surface Functional Groups and Compounds 

Fourier-transform infrared spectroscopy (FTIR) analysis was undertaken to verify the probable organic components in *Jatropha integerrima* extract responsible for AgNO_3_ reduction. FTIR spectra were recorded with SPECTRUM100, Perkin-Elmer, Wellesley, MA, USA, ranging between 450 and 3500 cm^−1^ utilizing a diffuse reflectance accessory, according to Siddiqi et al. [67]. 

### 4.7. Antibacterial Screening 

Antibacterial susceptibility assay was conducted by the agar well diffusion technique. Four human pathogenic bacteria were used to examine the antibacterial activity of AgNPs, two Gram-positive bacteria: methicillin-resistant *Staphylococcus aureus* (MRSA) and *Streptococcus mutans*, and two Gram-negative: *Klebsiella pneumoniae* and *Escherichia coli*. Bacteria were cultured from the Bio-house medical lab, Riyadh, Saudi Arabia. Nutrient agar medium (Oxoid) was used for the subculturing of each strain, and plates were incubated at 37 °C for 24 h. Bacterial suspensions in saline at a concentration of 1.5 × 10^8^ CFU/mL (McFarland standard, 0.5) were prepared using a naked eye after applying the direct colony suspension method. The plates were inoculated with the tested pathogenic strains, and 40 mL of J-AgNPs (1 mg/mL) were loaded separately into each well of the Petri plates and kept under aseptic conditions for 1 h for drying. Distilled water was treated as a negative control, and plates were incubated for 24 h at 37 °C according to the Clinical and Laboratory Standards Institute [70]. The clear area around the well was evaluated and expressed in mm.

### 4.8. Minimum Inhibitory Concentration and Minimum Bactericidal Concentration 

The inhibitory and bactericidal effects at their minimum concentrations of J-AgNPs were assessed using the serial dilution technique. A volume of 100 μL/well of bacterial suspensions at 0.5 McFarland standard were cultured in 96-well plates with varied levels of J-AgNPs concentrations (0.2, 0.5, 0.8, 1.1, 1.4, 1.7, 2.0, and 2.3 mg/mL) and then incubated at 37 °C for 24 h. MIC and MBC were identified by agar well diffusion test for the tested bacteria.

### 4.9. Ampicillin-Conjugated J-AgNPs (Amp-J-AgNPs) Synthesis, Analysis and Application

Amp-J-AgNPs nanocomposite was prepared by a minor change of the method reported by Fan et al. [40] using a one-pot reaction. First, 1 mL of ampicillin solution (1 mg/mL) was added to 1 mL of J-AgNPs (1 mg/mL). Next, the combination was shaken for 48 h at room temperature in a dark condition and then washed, and a solution of Amp-J-AgNPs (1 mg/mL) was prepared. Furthermore, the prepared nanocomposite was studied under TEM at 80 kV voltage, and the Amp-J-AgNPs size was measured using ImageJ software version 1.8.0 (National Institutes of Health, Bethesda, MD, USA). On the other hand, the antibacterial effects of the nanocomposite were studied via a standard well diffusion assay and evaluated by the diameters of the clear area around each well (mm) after 24 h of incubation at 37 °C. 

### 4.10. Anticancer Action and Apoptosis Induced by J-AgNPs

Cell viability assays were conducted using two breast cancer cell lines, MDA MBA 231 (established from a pleural effusion of a 51-year-old caucasian female with a metastatic mammary adenocarcinoma) [71] and HCT116 (human colorectal carcinoma cell line isolated from an adult male) [72], and one standard cell line MCF 10A (ATCC-CRL-10317) [71]. A 96-well plate was used for cell lines cultured at a 5 × 104 cells/well concentration and then kept at 37 °C and humidified air/CO_2_ at 95%/5%. Media was discharged after 24 h and replaced by phenol-red free DMEM, including fetal bovine serum FBS (0.5%) in cells. Cells were then treated with various concentrations of J-AgNPs and incubated for 48 h; then, the media was aspirated, and PBS was used for cell washing. Subsequently, the viability of the cell was verified by MTT assay according to the manufacturer’s protocol. In brief, a volume of 20 µL of 5 mg/mL MTT reagent was added to the cells and incubated at 37 °C for 4 h. After the supernatant was discharged, MTT formazan was dissolved in 100 µL dimethyl sulfoxide, and the absorbance was assessed at 570 nm by the molecular device spectra max microplate absorbance reader. Furthermore, to assess apoptosis, MDA MB 231 cells were examined by LSM and TEM analysis.

### 4.11. Cell Structural Changes by Transmission Electron Microscopy

MDA-MB-231 cells were seeded in plates (6 well), and J-AgNPs was added then kept for 24 h. Later, cells were collected, centrifuged, washed, and fixed for 2 h in glutaraldehyde (Product 16210, electron microscopy sciences (EMS), Hatfield, PA, USA) at a concentration of 4%, and then 1% osmium tetroxide ( Product 19100, EMS, Hatfield, PA, USA) was added for 1 h. Furthermore, 50%, 70%, and 100% ethanol were used for 10 min for dehydration. Then, for 15 min, samples were treated twice with 100% propylene oxide (Product 8.07027.1001, Merck KGaA, Darmstadt, Germany). Infiltration was made by a combination of EMbed 812 one-step single-mix formula composed of 20 mL of EMbed 812 (Product 14900, EMS, Hatfield, PA, USA), 16 mL of Dodecenyl Succinic Anhydride (DDSA) (Product 13710, EMS, Hatfield, PA, USA), 8 mL of Methyl-5-Norbornene-2,3-Dicarboxylic Anhydride (NMA) (Product 19000, EMS, Hatfield, PA, USA), and 0.66-0.88 mL of 2,4,6-Tri (dimethyl aminomethyl) phenol (DMP-30) (Product 13600, EMS, Hatfield, PA, USA). Samples were subsequently covered with a 1:1 solution of propylene oxide: embedding medium was kept for 1 h at room temperature (RT), and then embedding medium to propylene oxide (2:1) was added overnight. Lastly, a 100% embedding medium was added to the combination for 2 h at RT. Embedding was achieved by moving cells in EMS embedding capsules (Product 69910-05, EMS, Hatfield, PA, USA) before the embedding medium was loaded and kept for 24 h in the oven at 60 °C to produce blocks. Following cool down to RT, the blocks were manually cut, and 100–200 nm sections were obtained by an ultramicrotome (Product PT-PC #75840, RMC Boeckeler Instruments, Inc., Tucson, AZ, USA). After that, sections were loaded on a grid (Product G200-Cu, EMS), and 1% uranyl acetate (Product 93-2840, STREM CHEMICALS, Newburyport, MA, USA) was used for manual staining in the dark for 15 min, washed with normal saline 6 times, and then by 0.5% lead citrate (Product 17810, EMS, Hatfield, PA, USA); moreover, numerous pellets of sodium hydroxide were rinsed in distilled water. Following drying, samples were assessed by TEM (JEOL Ltd., Peabody, MA, USA).

### 4.12. Laser Scanning Microscopy (LSM)

MDA-MB-231 cells were seeded in an 8-well dish (Ibidi) 24 h before treatment. Cells were subjected to J-AgNPs (18 ug/mL) then kept at 37 °C for 24 h at CO_2_ (5%). Thereafter, cells were stained with Calcein AM (green) for live cells, HOECHST33342 (blue) for nucleus and PI (red) for dead cells. Images were acquired on Zeiss LSM780 microscope system using Argon laser at 488 nm/530 nm for Calcein AM, UV laser diode at 350 nm/460 nm for HOECST33342, and Intune laser at 490 nm/640 nm for PI. 

### 4.13. Statistical Analysis

MICROSOFT EXCEL 2019 was used for analyzing antibacterial results and expressed as means and standard deviations. One of the triplicates AgNPs images was chosen for illustration. GraphPad Prism Software version 9.1 (San Deigo, CA, USA) was used for statistical analysis.

## 5. Conclusions

In the current investigation, J-AgNPs were successfully synthesized using plant extract *J. integerrima*; its biological activity was demonstrated against four bacteria, two cancer cell lines and one normal cell line. J-AgNPs had a significant antibacterial effect; however, an antagonistic effect was noted when the Amp-J-AgNPs conjugate was tested, most likely due to the larger NP size and aggregation. Furthermore, J-AgNPs were cytotoxic to the tested cell lines to different degrees; this was confirmed based on the observed morphological changes that indicated the dispersion of J-AgNPs in the cytoplasmic membrane and other cell organelles, causing cellular damage via direct interaction between J-AgNPs and cellular components. In conclusion, the present study could be promising for delivering ecofriendly NPs as antibacterial and anticancer therapies. Furthermore, the well-known toxicity associated with AgNPs is expected to be reduced in the adopted, biological synthesis approach. However, further investigations are required to validate this presumption. A thorough examination of the synergistic ability of the antibiotic nanocomposite is required to increase the antibiotic effect against bacteria and mitigate MDR organisms. The current study adds to the body of the literature that already exists in the field of green synthesized AgNP and their use. It is important to note that this is the first study producing AgNPs from the plant *J. integerrima* and testing their biological effects. In addition, we lay the foundation for further studies to investigate the safety and therapeutic potential of J-AgNPs as an antimicrobial and anticancer agent.

## Figures and Tables

**Table 1:** Minimum inhibitory concentration (MIC) and minimum bactericidal concentration (MBC) of J-AgNPs against tested bacteria.

Microbes	MIC (mg/mL)	MBC (mg/mL)	MIC/MBC
*S. aureus*	0.8	1.1	0.72
*S. mutans*	0.8	1.1	0.72
*E. coli*	1.1	1.4	0.78
*K. pneumoniae*	1.1	1.4	0.78

## Data Availability

Data of this manuscript are displayed in Figure 1, Figure 2, Figure 3, Figure 4, Figure 5, Figure 6, Figure 7, Figure 8, Figure 9, Figure 10, Figure 11 and Figure 12. The facts and raw data analyzed are available from the corresponding author upon request.
